# Simulated virtual on-call training programme for improving non-specialised junior doctors' confidence in out-of-hours psychiatry: quantitative assessment

**DOI:** 10.1192/bjb.2022.40

**Published:** 2023-10

**Authors:** Helen Blamey, Charlotte H. Harrison, Alistair Roddick, Tina Malhotra, Kate E. A. Saunders

**Affiliations:** 1 John Radcliffe Hospital, Oxford, UK; 2 Oxford University Clinical Academic Graduate School, John Radcliffe Hospital, Oxford, UK; 3 Littlemore Health Centre, Oxford, UK; 4 Department of Psychiatry, University of Oxford, Warneford Hospital, Oxford, UK

**Keywords:** Cost-effectiveness, education and training, information technologies, virtual training, simulation

## Abstract

**Aims and method:**

To investigate whether a psychiatry-specific virtual on-call training programme improved confidence of junior trainees in key areas of psychiatry practice. The programme comprised one 90 min lecture and a 2 h simulated on-call shift where participants were bleeped to complete a series of common on-call tasks, delivered via Microsoft Teams.

**Results:**

Thirty-eight trainees attended the lecture, with a significant improvement in confidence in performing seclusion reviews (*P* = 0.001), prescribing psychiatric medications for acute presentations (*P* < 0.001), working in section 136 suites (places of safety) (*P* = 0.001) and feeling prepared for psychiatric on-call shifts (*P* = 0.002). Respondents reported that a virtual on-call practical session would be useful for their training (median score of 7, interquartile range 5–7.75). Eighteen participants completed the virtual on-call session, with significant improvement in 9 out of the 10 tested domains (*P* < 0.001).

**Clinical implications:**

The programme can be conducted virtually, with low resource requirements. We believe it can improve trainee well-being, patient safety, the delivery of training and induction of rotating junior doctors during the COVID-19 pandemic and it supports the development and delivery of practical training in psychiatry.

Out-of-hours shifts (‘on-call’ shifts) are an aspect of clinical practice for which newly appointed junior doctors feel unprepared and are frequently associated with negative emotional responses.^1^ Out-of-hours work is associated with subjective lack of support and increased workload and is perceived by junior doctors to be a strong factor contributing to prescription errors.^2,3^ Medical staffing in psychiatry is often reduced outside of normal working hours, and thus the impact on junior doctors staffing the out-of-hours rota is significant. There is less access to senior support, and junior staff are called to respond first to any psychiatric emergencies.^4^ Additionally, psychiatry is often taught in the middle of the clinical course during medical school, so most junior doctors will not have had recent psychiatric experience when they start their psychiatry job.

Simulated on-call programmes provide an opportunity to train medical students and junior doctors to develop skills relevant to out-of-hours working that are not generally covered in standard medical curricula, such as task prioritisation and interpersonal communication.^4^ By simulating the type, frequency and complexity of tasks expected during on-call shifts, these programmes aim to improve junior doctors’ familiarity and confidence in working out of hours. Such programmes have been undertaken in the context of general medical and surgical on-call shifts, resulting in improved confidence among participants.^5,6^ Psychiatry on-call shifts, however, differ considerably from medical or surgical on-calls; they require a different skill set, knowledge base and approach to time management (owing to cross-site or non-resident cover). High-fidelity simulation sessions of psychiatric cases have shown effectiveness in improving confidence of trainees.^4^ Despite this, few virtual on-call programmes exist in psychiatry to develop the specific skills described above and, crucially, to improve confidence in out-of-hours practice.

Here we present an evaluation of psychiatry-specific virtual on-call programme for non-specialised junior doctors which has been running at Oxford Health NHS Foundation Trust.

## Method

**Fig. 1:**
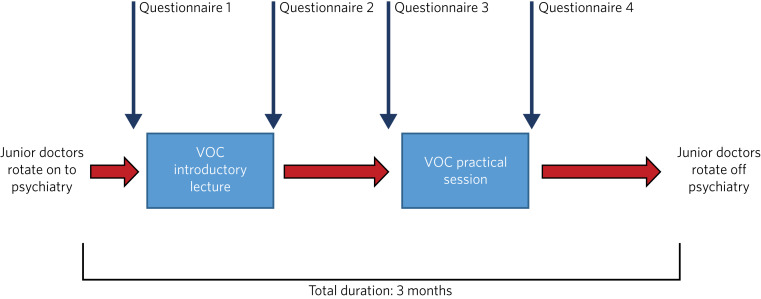
Evaluation flow diagram. VOC, virtual on-call.

The virtual on-call programme commenced in December 2020, in line with the rotation of foundation doctors into psychiatry posts within Oxford Health NHS Foundation Trust. It consisted of a virtual 2 h introductory lecture, followed by a 2 h simulated on-call shift (Fig. 1).

The introductory lecture was delivered by three authors (H.B., C.H., A.R.) using PowerPoint, via Microsoft Teams, on the first week of the psychiatry rotation, during the trust induction for new doctors. This lecture described the format of the virtual on-call programme, the objectives of the sessions, dates of sessions and how to sign up. It provided general information that would be required for the completion of scenario tasks in the practical sessions, for example information on trust guidelines (e.g. on rapid tranquillisation, nicotine replacement therapy, correcting electrolyte derangements) and how to access them, how to perform a seclusion review, how to complete a Mental Health Act section 5(2) form,^2^ information on the section 136 suites (places of safety), how to complete medical or surgical referrals for patients admitted to the psychiatric wards and how to conduct an admission clerking.

Following this, a series of 2 h simulated on-call shifts were hosted for two participants per session once a week for 10 weeks. All trainees were offered the opportunity to attend during their attachment. This included Foundation Year (FY) 1 and 2 doctors and General Practice Vocational Training Scheme (GPVTS) trainees. The FY2 and GPVTS trainees contribute to the same on-call rota and hence require the same on-call training. The programme included FY1 doctors, as scenarios reflected in-hours tasks that FY1 doctors could be required to attend, despite not participating in the on-call rota. Each session was run by an FY2 doctor who had completed a psychiatry post within the trust and by a psychiatry core trainee doctor in year 2 or above (CT2+), who simulated the role of the psychiatry registrar/senior support. The trust's medical education lead (a consultant psychiatrist) reviewed the teaching materials prior to the sessions and the medical education administration team assisted with organising session attendance.

During the session, trainees were ‘bleeped’ with different psychiatry-specific tasks (Table 1). The scenarios were designed to reflect common on-call tasks encountered during out-of-hours work. A full illustration of the scenarios is given in Appendix 1.

**Table 1:** Scenarios included in the virtual on-call practical session

Scenario 1	Rapid tranquillisation
Scenario 2	Admission clerking
Scenario 3	Seclusion review
Scenario 4	Mental Health Act section 5(2)
Scenario 5	Management of paracetamol overdose
Scenario 6	Delirium secondary to urosepsis
Scenario 7	Assessment of physical injury
Scenario 8	Nicotine replacement prescription for patient on clozapine who has stopped smoking
Scenario 9	Alcohol withdrawal
Scenario 10	Refeeding syndrome and electrolyte replacement

Participants were provided with a scenario brief. They had access to mocked-up patient information, such as medical and psychiatric history, drug chart and regular medication, psychiatric diagnosis(es) and admission status. The participants were then required to engage in further history taking, which was done as role-play conversationally with the session facilitator(s), and to describe their initial investigations or management once they had gathered sufficient information. If the scenario required physical assessment, such as scenario 7, then physiological parameters such as observations or blood test results were provided. Participants worked through the tasks independently, using local intranet policies and telephone advice from the core trainee in the role of the on-call psychiatry registrar for any management queries.

Following completion of scenarios, there was a debriefing, with the opportunity for feedback and questions.

Participants completed a series of anonymous questionnaires evaluating their confidence in ten domains, rated on a Likert scale from 0 to 10. Questionnaires were completed at four time-points during the programme: pre- and post-introductory lecture and pre- and post-simulated shift (Fig. 1). Scores were compared using Mann–Whitney U non-parametric tests. Significance was defined as *P* < 0.05, with Bonferroni correction applied for multiple testing.

This service evaluation was approved by Oxford Health NHS Foundation Trust. Approval was given by Oxford Health NHS Foundation Trust to conduct this service evaluation (audit number = 185). Data was collected anonymously and thus consent was not collated from participants. Participants were informed of the intended use and anonymity of data prior to data collection.

## Results

### Pre- and post-session questionnaires: virtual introductory lecture

The initial pre-lecture questionnaire was sent out to 38 doctors in training. Respondents completed the questionnaire at the start of their psychiatry rotation. All 38 returned responses (14 FY1 doctors, 12 FY2 doctors, and 12 GPVTS (Specialty Trainee Year 1) trainees).

Of the 38 respondents, 28 reported less than 2 months of psychiatry experience (including during medical school) and 10 reported less than 1 month of psychiatry experience. Nine of the respondents reported previous experience of simulated on-call programmes, although none reported such teaching in psychiatry-specific contexts.

**Fig. 2:**
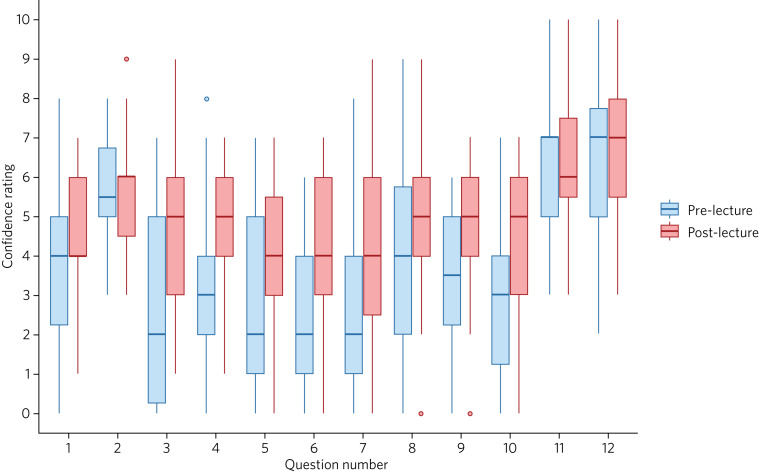
Box plot showing questionnaire results before and after the introductory lecture. Circles represent outlier values. Light grey indicates pre-lecture responses and dark grey indicates post-lecture responses. Question numbers correspond to questions listed in Table 2.

Self-reported confidence was low (≤5) across all assessed domains (Table 2 and Fig. 2), with respondents reporting the lowest confidence in performing seclusion reviews (median score 2; interquartile range (IQR) 0.25–5), detention of patients under the Mental Health Act (median score 2; IQR 1–5), in management of patients in section 136 suites (median score 2; IQR 1–4) and in working cross-site during out-of-hours shifts (median score 2; IQR 1–4). Respondents reported that a psychiatry virtual on-call practical session would be useful in their training, with a median score of 7 (IQR 5–7.75).

**Table 2:** Self-reported confidence before and after the psychiatry virtual on-call lecture^a^

Question number	Question	Pre-lecture confidence (*n* = 38)	Post-lecture confidence (*n* = 31)	*P*
1	How confident do you feel in assessing patients with acute psychiatric conditions?	4 (2.25−5)	4 (4−6)	0.11
2	How confident do you feel assessing and managing physical health presentations on the psychiatric wards?	5.5 (5−6.75)	6 (4.5−6)	0.90
3	How confident do you feel performing seclusion reviews?	2 (0.25−5)	5 (3−6)	0.001*
4	How confident do you feel prescribing psychiatric medications for acute psychiatric presentations?	3 (2−4)	5 (4−6)	<0.001*
5	How confident are you in detaining patients under relevant sections of the Mental Health Act?	2 (1−5)	4 (3−5.5)	0.0043
6	How confident are you working with patients in the place of safety section 136 suites?	2 (1−4)	4 (3−6)	0.001*
7	How confident are you working cross-site during out-of-hours shifts?	2 (1−4)	4 (2.5−6)	0.005
8	How confident are you using the virtual handover system for psychiatry within Oxford Health?	4 (2−5.75)	5 (4−6)	0.077
9	Overall, how confident do you feel in managing acute psychiatric presentations?	3.5 (2.25−5)	5 (4−6)	0.006
10	How prepared do you feel for psychiatric on-call shifts?	3 (1.25−4)	5 (3−6)	0.002*
11	How useful do you feel that the virtual on-call training lecture will be for your learning?	7 (5−7)	6 (5.5−7.5)	0.999
12	How useful do you feel that the virtual on-call practical session will be for your learning?	7 (5−7.75)	7 (5.5−8)	0.67

a. Confidence ratings were scored on a scale of 0 to 10 and are displayed as median (interquartile range). Results were considered significant at *P* < 0.0042.

* *P* < 0.0042 (i.e. with Bonferroni correction applied).

The post-lecture questionnaire was completed by 31 participants (response rate 81.5%). Participants reported a significant improvement in confidence in performing seclusion reviews (*P* = 0.001), prescribing psychiatric medications for acute psychiatric presentations (*P* < 0.001), working with patients in the section 136 suites (*P* = 0.001) and in feeling of preparedness for psychiatric on-call shifts (*P* = 0.002).

### Pre- and post-session questionnaires: simulated on-call session

**Fig. 3:**
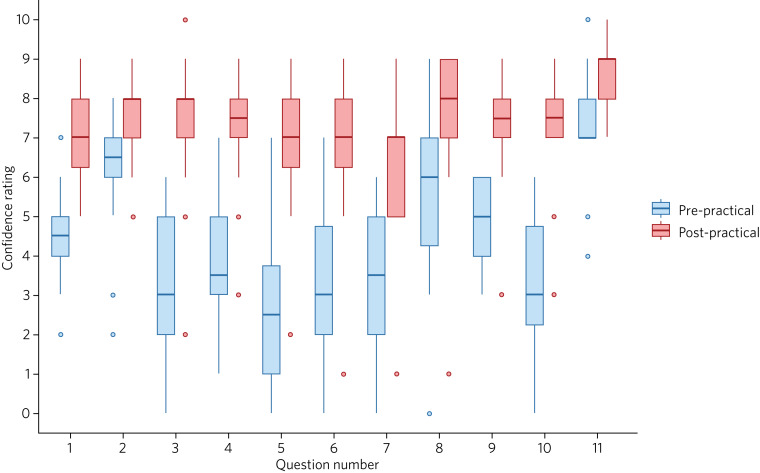
Box plot showing questionnaire results before and after the practical session. Circles represent outlier values. Light grey indicates pre-session responses and dark grey indicates post-session responses. Question numbers correspond to questions listed in Table 3.

Eighteen participants completed the virtual on-call session and all 18 returned questionnaire responses (11 FY1 doctors, 4 FY2 doctors and 3 core trainee doctors). Participants reported a significant improvement in nine out of the ten tested domains (Table 3 and Fig. 3). The only area where there was not a significant improvement in pre- and post-session rating was the use of the virtual handover system (*P* = 0.005).

**Table 3:** Self-reported confidence before and after the psychiatry virtual on-call (VOC) practical session^a^

Question number	Question	Pre-VOC confidence (*n* = 18)	Post-VOC confidence (*n* = 18)	*P*
1	How confident do you feel in assessing patients with acute psychiatric conditions?	4.5 (4−5)	7 (6.25−8)	<0.001*
2	How confident do you feel assessing and managing physical health presentations on the psychiatric wards?	6.5 (6−7)	8 (7−8)	0.002*
3	How confident do you feel performing seclusion reviews?	3 (2−5)	8 (7−8)	<0.001*
4	How confident do you feel prescribing psychiatric medications for acute psychiatric presentations?	3.5 (3−5)	7.5 (7−8)	<0.001*
5	How confident are you in detaining patients under relevant sections of the Mental Health Act?	2.5 (1−3.75)	7 (6.25−8)	<0.001*
6	How confident are you working with patients in the place of safety section 136 suites?	3 (2−4.75)	7 (6.25−8)	<0.001*
7	How confident are you working cross-site during out-of-hours shifts?	3.5 (2−5)	7 (5−7)	<0.001*
8	How confident are you using the virtual handover system for psychiatry within Oxford Health?	6 (4.25−7)	8 (7−9)	0.005
9	Overall, how confident do you feel in managing acute psychiatric presentations?	5 (4−6)	7.5 (7−8)	<0.001*
10	How prepared do you feel for psychiatric on-call shifts?	3 (2.25−4.75)	7.5 (7−8)	<0.001*
11	How useful do you feel that the virtual on-call practical session will be for your learning?	7 (7−8)	9 (8−9)	0.001*

a. Confidence ratings were scored on a scale of 0 to 10 and are displayed as median (interquartile range). Results were considered significant at *P* < 0.0045.

* *P* < 0.0045 (i.e. with Bonferroni correction applied).

## Discussion

This evaluation demonstrates that the virtual on-call training programme improved participants’ confidence in multiple aspects of psychiatric out-of-hours practice. Although both the lecture session and the practical session led to improvements in confidence, the practical session showed statistically significant improvements in nine out of ten domains, and was thus more effective at increasing confidence than the lecture session, which showed statistically significant improvements in four out of ten domains. Trainees undertaking the sessions reported feeling more prepared for psychiatric out-of-hours work following both sessions. We believe this virtual on-call programme is a pragmatic and low-cost intervention that contributes to the development of confidence in common on-call tasks and feeling prepared for out-of-hours work among non-specialised junior doctors.

### Near-peer and peer-to-peer teaching

The benefits of near-peer teaching in medical education are widely recognised.^7–9^ The virtual on-call programme uses a combination of both near-peer (the CT2+ trainee in the role of senior support and the learner)^10^ and peer-to-peer teaching (session facilitator and learner) to run both the scenarios and the debriefings. We believe some of the educational value of this intervention results from social and cognitive congruence: unique underlying semantic networks shared by the near-peer teacher and the learner.^11^

Cognitive congruence results from the recency of the near-peer teacher's primary learning experience, allowing them to clearly explain complex topics and emphasise key points for the learner.^9,11,12^ In our experience, much of the value of simulation is transitioning the ‘know how’ into the ‘how to’, rather than introducing new complex concepts. Therefore, the proximity of the facilitators to their on-call work allows them to relay to the learners/attendees the key skills that they learned so recently. Social congruence is the alignment of the learner's and the facilitator's attitudes to learning, motivation and well-being.^11,12^ This may be particularly relevant for the anxiety and anticipation associated with on-call shifts, which can only be alleviated by someone with local, recent experience of the same work – as per our programme.

Debriefing is integral to maximising the educational value of simulation-based training.^13^ There are many questions in the medical education literature about how to achieve the optimal debriefing, including who should facilitate it.^14^ This last question has been explored in the emergency medicine simulation setting. Cooper et al compared resident-led debriefings with faculty-led debriefings on Debriefing Assessment for Simulation in Healthcare (DASH) scores. They found no significant difference in DASH scores and concluded that resident-led debriefings were as effective as faculty-led debriefings.^15^ Similarly, we did not use faculty staff or senior educators, despite the additional educational experience they may bring. Although our evaluation was not designed to explore this comparison, the average score from attendees in the post-session survey for the statement ‘The facilitator improved my knowledge’ was 8.9/10 (data not shown). This high score suggests that the lack of facilitator seniority was not perceived as a disadvantage. There are additional practical benefits to using FY2 and CT2 trainees as facilitators, including greater availability and lower cost. This allowed learners to attend in pairs, rather than small groups, so that they received more individualised attention. Future iterations of the project could seek to compare the efficacy of debriefings led by senior versus junior educators, as identified by Raemer et al.^14^

### Trainee well-being

Improved self-confidence and feelings of readiness for out-of-hours work has a positive impact on trainee well-being. On average, participants in our programme indicated an improvement in feelings of preparedness for out-of-hours work, thus reducing the burden of anxiety regarding these shifts. On-call work commonly results in increased stress for trainees due to unpredictability of tasks,^16^ and programmes such as the psychiatry virtual on-call programme described here can help mitigate this through task exposure in a safe environment. A 2017 systematic review by Alanazi et al indicates that the development of participant confidence in turn helps trainees to develop skills and acquire knowledge;^17^ thus the symbiosis of confidence and learning is mutually beneficial.

### COVID-19 impact on junior doctor changeover

The COVID-19 pandemic has had a significant impact on postgraduate training,^18–20^ and psychiatry is no exception.^21^ Rotation on to new specialties by foundation doctors is perceived to be a stressful time of feeling underprepared and unsupported.^22,23^ With loneliness and isolation occurring during a socially distanced pandemic,^24^ particularly in specialties that readily adopt remote working, such as psychiatry, trainees may associate changeover with increased anxiety and uncertainty. As a result, it is especially important to ensure that additional strategies are in place to help transition between rotations.

Other groups have used structured teaching^23^ or simulated on-call sessions^25^ around the changeover period in non-psychiatry specialties, resulting in significantly increased confidence and knowledge. A virtual on-call programme for final year medical students run by Stone et al helped improve students’ confidence and preparedness for practice.^26^ Our evaluation supports the observation that targeted training during the induction period can improve confidence in new trainees, and furthers the field by applying this to a psychiatry-specific setting. We believe our intervention would be of benefit to other psychiatry foundation trusts looking for a low-cost, accessible, effective intervention to improve confidence in non-specialised medical trainees.

### Lecture-based versus practical teaching

Although our evaluation was not designed to compare didactic teaching (the virtual lecture) with practical (the virtual on-call shift), we find that improvements in knowledge were greater after the on-call shift than the lecture (Figs 1, 2). Medical education literature suggests that simulation can be more effective than lecture-based teaching. A meta-analysis of 17 studies comparing simulation in the critical care setting with other teaching modalities found that simulation was more effective at improving performance-related outcomes.^27^ It did not find evidence that simulation was better at preparing for knowledge-based assessments – supporting the theory that simulation is helpful for converting ‘book knowledge’ into ‘practical knowledge’. Our data are consistent with this idea, of helping trainees convert information learned about psychiatry at medical school into practical skills for applying psychiatry in practice out of hours. In addition, Semler et al randomised medical interns to didactic, demonstration and simulation teaching.^28^ They found that teamwork skills were improved significantly following demonstration-based or simulation teaching, compared with didactic teaching, despite clinical performance scores being similar.^28^ This highlights how essential non-practical skills are not best taught through didactic learning. With no studies known to us comparing didactic with practical teaching in the psychiatry setting, future studies should explore this further.

One major drawback of simulation learning is that it is a costly technique – intensive on resources and staffing.^27,29,30^ The psychiatry virtual on-call programme offers the benefits of simulating an on-call shift, but with low resource requirements – the programme was run over Microsoft Teams, materials were disseminated online and sessions took place during protected time for trainees to attend or deliver teaching.

### Patient safety

The role of simulation in improving patient safety is increasingly well evidenced in the literature. Simulated scenarios are a powerful tool in the education spectrum that can contextualise learning and awareness of competencies, thus having potential to improve patient safety through learning new strategies for safe and effective practice.^31,32^ Although we did not measure patient safety outcomes, we hope that improving training and perceived confidence could translate to clinical benefit. Future work to appraise the longer-term impact of this intervention would aim to evaluate patient safety metrics.

### Limitations

This evaluation has several limitations. It was limited to one cycle of participants at various stages of training, with a modest sample size of 18 for the simulated on-call session. However, despite the limited sample size, robust differences in confidence were detectable after both the lecture and practical sessions. Larger samples might permit further analysis of improvements stratified by training grade.

The surveys were conducted anonymously, preventing paired analysis of pre- and post-session questionnaires. We were therefore unable to identify the number of trainees exhibiting positive responses to the programme or to track individuals’ changes in confidence over time. Such analyses might offer a more robust comparison of the didactic and simulated elements of the programme. Furthermore, we were unable to assess whether transient improvements in confidence immediately following the sessions were maintained over the longer term. In future iterations of the project, a final questionnaire at the end of the junior doctors’ rotation might be beneficial to reassess confidence and to assess perceived utility of the project.

As this was a service evaluation there was no randomisation of participants, as participants were already allocated to the general adult psychiatry rotation as part of their training programme.

There is potential for selection bias, given that fewer trainees attended the practical sessions than attended the introductory lecture. It is possible that the participants who prioritised attending a practical session were those who felt least confident in out-of-hours work.

Owing to COVID-19, the programme had to be rapidly adapted to online delivery. Although this adaptation worked well for the delivery of the lecture-based component, there was a considerable loss of fidelity for the practical component and a loss of independence for participants when working through the scenarios. When it is possible to return to face-to-face small-group teaching, it would be of benefit to deliver this programme in its originally planned offline format. Conversely, the online format led to additional advantages, including greater flexibility for the facilitating staff and reduced need for venues.

As this was a pilot programme, we used a quantitative assessment method. This allowed early objective detection of increased confidence following the intervention and justified continued implementation in the curriculum. Thomson et al (2013)^4^ used qualitative analysis to identify the key themes that made their simulated psychiatry emergency training effective. In future, we (and indeed any other groups considering implementing our virtual on-call programme) should consider using focus groups and/or semi-structured interviews for a more meaningful understanding of the benefits to the learner.

Despite these limitations we were still able to see statistically significant improvements in participants’ confidence across the tested domains as a result of the virtual on-call session.

### Conclusions

The psychiatry virtual on-call programme had clear benefits for participating non-specialised junior doctors who were commencing a psychiatry rotation in Oxford Health NHS Foundation Trust. It improved confidence and readiness for out-of-hours working and was perceived by trainees to be a useful learning experience. The programme was successfully delivered online, reducing the cost and use of physical resources and enabling easier access to the programme, given the cross-site and community job roles that trainees are employed in. We propose that this simulated on-call programme is an important addition to the induction of non-specialised junior doctors expected to fulfil out-of-hours work in psychiatry and could be implemented more widely across NHS trusts in the UK.

## Data Availability

The data that support the findings of this evaluation are available from the corresponding author (H.B.) on reasonable request.

## About the authors

**Helen Blamey** is a Foundation Year 2 Doctor at the Oxford University Clinical Academic Graduate School, John Radcliffe Hospital, Oxford Health NHS Foundation Trust, Oxford, UK. **Charlotte H. Harrison** is a Foundation Year 2 Doctor at the Oxford University Clinical Academic Graduate School, John Radcliffe Hospital, Oxford Health NHS Foundation Trust, Oxford, UK. **Alistair Roddick** is a Foundation Year 2 Doctor at the Oxford University Clinical Academic Graduate School, John Radcliffe Hospital, Oxford Health NHS Foundation Trust, Oxford, UK. **Tina Malhotra** is a Consultant Psychiatrist at Littlemore Health Centre, Oxford Health NHS Foundation Trust, Oxford, UK. **Kate E. A. Saunders** is an Associate Professor in the Department of Psychiatry, Oxford University Clinical Academic Graduate School, John Radcliffe Hospital, Oxford Health NHS Foundation Trust, Oxford, UK, and in the Department of Psychiatry, University of Oxford, Warneford Hospital, Oxford, UK.

## Appendix

**Scenario 1**

Brief: You have been called by a nurse on X ward, who is concerned about a patient's level of agitation. They have tried verbal de-escalation to little effect, and they are wondering whether you could attend to assess the patient and consider prescribing medication.

Setting: In-patient general adult psychiatry ward.

Participants: 2 junior doctors, 1 facilitator (performing role-play of agitated patient), 1 psychiatry core trainee/specialty registrar providing advice.

Aids provided: Drug chart; link to Oxford Health Foundation Trust guideline on rapid tranquillisation.

Learning objectives: How to manage agitated patient – non-pharmacological and pharmacological management; how to use trust guideline and prescribe medication safely and accurately.

**Scenario 2**

Brief: You are the day duty doctor on call for the Warneford Hospital. You have just received a bleep from the ward FY1 on Vaughan Thomas ward. It is his first day on the ward, and his SHO, SpR and consultant have been called away. A new patient has arrived and needs an admission clerking, but he is unsure how to do this. Please talk him through the relevant steps of an admission clerking.

Participants: 2 junior doctors, 1 psychiatry core trainee/specialty registrar providing advice.

Aids: Nil.

Learning objectives: How to perform psychiatric admission clerking, prescribing of regular medications and discretionary medications, physical examination and site-specific requirements such as VTE risk assessment, COVID swabs.

**Scenario 3**

Brief: You have been called by one of the nursing team from Ashurst Ward (PICU) as they have just had to put a patient in seclusion and require a medical review as per seclusion protocol. He was put into seclusion after starting a fight with another patient and punching him in the face.

Participants: 2 junior doctors, 1 facilitator (performing role-play of agitated patient), 1 psychiatry core trainee/specialty registrar providing advice.

Aids: Nil.

Learning objectives: How to perform seclusion review, including correct documentation; timelines for seclusion reviews.

**Scenario 4**

Brief: You have been called by one of the nurses on Wintle Ward. There is a patient on an informal admission who is requesting unaccompanied leave, but the team are concerned about her level of risk. The patient is a 24-year-old woman who was admitted with a diagnosis of severe depression 10 days ago. Please assess the patient and make an appropriate management plan.

Participants: 2 junior doctors, 1 facilitator (performing role-play of patient), 1 psychiatry core trainee/specialty registrar providing advice.

Aids: Form H1 (section 5(2) form).

Learning objectives: Risk assessment, awareness of informal admission status, 5(2) paperwork completion and correct filing of this, next steps after completing 5(2), e.g. MHAA.

**Scenario 5**

Brief: A 27-year-old patient admitted to the adult psychiatry ward under a section 2 with a diagnosis of severe depression has informed the nursing staff that she took a paracetamol overdose during her unaccompanied leave 4 hours ago. The nursing staff have taken bloods, including a paracetamol level, and want you to review the results and advise any further management. Is there any additional information you would like? Please describe your next steps and actions.

Participants: 2 junior doctors, 1 facilitator, 1 psychiatry core trainee/specialty registrar providing advice.

Aids: Mocked-up blood results, including paracetamol level (high), liver function tests (high ALT), clotting; TOXBASE paracetamol management guideline, including nomogram.

Learning objectives: How to take a history in drug overdose, how to access TOXBASE and use paracetamol nomogram, how to treat paracetamol overdose (*N*-acetylcysteine), requirements for transfer out of psychiatry hospital for intravenous therapy.

**Scenario 6**

Brief: The nursing staff on the old age psychiatry ward have asked you to review an 83-year-old patient admitted with Lewy body dementia. He has become increasingly agitated and is reporting new hallucinations. They are wondering whether he requires some sedation to help calm him down.

Participants: 2 junior doctors, 1 facilitator (performing role-play of nurse), 1 psychiatry core trainee/specialty registrar providing advice.

Aids: Observations (pyrexia, tachycardia), examination report.

Learning objectives: Identification of physical cause for deterioration, likely delirium secondary to infection, need for acute management and how to access medical services (transfer to acute hospital).

**Scenario 7**

Brief: A 23-year-old patient is admitted under a section 2 following a manic episode (known BPAD). After an altercation with another patient earlier she became agitated and aggressive. The situation was de-escalated and she was encouraged to go to her room, where she started headbanging against the wall. She was restrained by the nursing staff using safe restraint until she calmed down. She remains calm but has an obvious wound on her forehead, which the nursing staff would like you to review. Please give your response and describe your next steps.

Participants: 2 junior doctors, 1 facilitator (performing role-play of nurse), 1 psychiatry core trainee/specialty registrar providing advice.

Aids: Photograph of superficial abrasion to forehead, examination reports (physical and neurological examination).

Learning objectives: How to assess headbanging injuries, wound assessment, NICE CT head guidelines, understanding pathways to facilitate investigations if required.

**Scenario 8**

Brief: You have been asked by a nurse on Allen Ward to prescribe nicotine replacement therapy (NRT) for a patient who was admitted 2 days ago. The patient has a long-standing diagnosis of schizophrenia and is managed on clozapine in the community. She was admitted with an increase in paranoid thoughts and general deterioration in function.

Participants: 2 junior doctors, 1 facilitator (performing role-play of nurse), 1 psychiatry core trainee/specialty registrar providing advice.

Aids: Trust guideline on NRT, NRT prescription chart.

Learning objectives: Need for awareness of smoking status and NRT prescribing on admission, correct mode of prescribing, awareness of impact of smoking on clozapine levels.

**Scenario 9**

Brief: You have been called by the nursing staff on Ashurst Ward regarding a patient admitted to the 136 suite. The patient is a 56-year-old male who was found with suicidal intent on the roof of a multi-storey building. They have limited history but are aware he has a history of alcohol excess and are concerned he might be at risk of alcohol withdrawal. Please assess the patient and form a management plan.

Participants: 2 junior doctors, 1 facilitator (performing role-play of nurse), 1 psychiatry core trainee/specialty registrar providing advice.

Aids: Trust guideline on managing alcohol withdrawal, prescription chart, observations (tachycardia).

Learning objectives: How to take an alcohol history (e.g. volume, timing of last drink, previous withdrawal seizures) and important examination features (e.g. agitation, tachycardia, tremor); use of scoring systems, e.g. CIWA; correct prescription, e.g. chlordiazepoxide weaning regimen, vitamin supplementation; awareness of risks, e.g. seizures.

**Scenario 10**

Brief: You have been called by the nursing staff at Cotswold House (eating disorder unit), asking you to review the routine refeeding bloods taken for a new patient admitted yesterday with a diagnosis of anorexia nervosa. She was started on a feeding regimen yesterday on admission. Please check the blood results and describe your management for any abnormalities you identify.

Participants: 2 junior doctors, 1 facilitator (performing role-play of nurse), 1 psychiatry core trainee/specialty registrar providing advice.

Aids: Blood results (mild low potassium, mild low phosphate), trust guideline on electrolyte replacement.

Learning objectives: Awareness of risk of refeeding syndrome in this patient group, correct prescription of electrolyte replacement as per trust guideline.

## References

[1] Goldacre MJ, Lambert T, Evans J, Turner G. Preregistration house officers’ views on whether their experience at medical school prepared them well for their jobs: national questionnaire survey. BMJ 2003; 326: 1011–2. 12742922 10.1136/bmj.326.7397.1011 PMC154758

[2] Lewis PJ, Ashcroft DM, Dornan T, Taylor D, Wass V, Tully MP. Exploring the causes of junior doctors’ prescribing mistakes: a qualitative study. Br J Clin Pharmacol 2014; 78: 310–9. 24517271 10.1111/bcp.12332 PMC4137823

[3] Alanazi MA, Tully MP, Lewis PJ. Prescribing errors by junior doctors: a comparison of errors with high risk medicines and non-high risk medicines. PLoS One 2019; 14(1): e0211270. 30703104 10.1371/journal.pone.0211270 PMC6355202

[4] Thomson AB, Cross S, Key S, Jaye P, Iversen AC. How we developed an emergency psychiatry training course for new residents using principles of high-fidelity simulation. Med Teach 2013; 35: 797–800. 24006955 10.3109/0142159X.2013.803522

[5] McGlynn MC, Scott HR, Thomson C, Peacock S, Paton C. How we equip undergraduates with prioritisation skills using simulated teaching scenarios. Med Teach 2012; 34: 526–9. 22452281 10.3109/0142159X.2012.668235

[6] Ramsden N, Newman J, Cooper R, Wilson A. ‘An hour on call’ – simulated medical education. Future Hosp J 2016; 3(suppl 2): s41. 31098270 10.7861/futurehosp.3-2s-s41 PMC6465916

[7] Ten Cate O, Durning S. Peer teaching in medical education: twelve reasons to move from theory to practice. Med Teach 2007; 29: 591–9. 17922354 10.1080/01421590701606799

[8] ten Cate O, van de Vorst I, van den Broek S. Academic achievement of students tutored by near-peers. Int J Med Educ 2012; 3: 6–13.

[9] Rees EL, Quinn PJ, Davies B, Fotheringham V. How does peer teaching compare to faculty teaching? A systematic review and meta-analysis. Med Teach 2016; 38: 829–37. 26613398 10.3109/0142159X.2015.1112888

[10] Whitman NA. Peer Teaching: To Teach Is to Learn Twice. ASHE-ERIC Higher Education Report No. 4. Washington, D.C.: Association for the Study of Higher Education, 1988.

[11] Moust JH, Schmidt HG. Facilitating small-group learning: a comparison of student and staff tutors’ behavior. Instr Sci 1994; 22: 287–301.

[12] Lockspeiser TM, O'Sullivan P, Teherani A, Muller J. Understanding the experience of being taught by peers: the value of social and cognitive congruence. Adv Health Sci Educ Theory Pract 2008; 13: 361–72. 17124627 10.1007/s10459-006-9049-8

[13] McGaghie WC, Issenberg SB, Petrusa ER, Scalese RJ. A critical review of simulation-based medical education research: 2003–2009. Med Educ 2010; 44: 50–63. 20078756 10.1111/j.1365-2923.2009.03547.x

[14] Raemer D, Anderson M, Cheng A, Fanning R, Nadkarni V, Savoldelli G. Research regarding debriefing as part of the learning process. Simul Healthcare 2011; 6(7): S52–7. 10.1097/SIH.0b013e31822724d0 21817862

[15] Cooper DD, Wilson AB, Huffman GN, Humbert AJ. Medical students’ perception of residents as teachers: comparing effectiveness of residents and faculty during simulation debriefings. J Grad Med Educ 2012; 4: 486–9. 24294426 10.4300/JGME-D-11-00269.1 PMC3546579

[16] Ziebertz CM, van Hooff ML, Beckers DG, Hooftman WE, Kompier MA, Geurts SA. The relationship of on-call work with fatigue, work-home interference, and perceived performance difficulties. Biomed Res Int 2015; 2015: 643413. 26558276 10.1155/2015/643413 PMC4628979

[17] Alanazi AA, Nicholson N, Thomas SL. The use of simulation training to improve knowledge, skills, and confidence among healthcare students: a systematic review. Internet J Allied Health Sci Pract 2017; 15(3): Art 2.

[18] Fonseka TR, Ellis RJ. The personal impact of COVID-19 on trainees. BMJ 2020; 371: m4688. 33262114 10.1136/bmj.m4688

[19] Seifman MA, Fuzzard SK, To H, Nestel D. COVID-19 impact on junior doctor education and training: a scoping review. Postgrad Med J 2022; 98: 466–76. 33688067 10.1136/postgradmedj-2020-139575

[20] General Medical Council. National Training Survey 2020: Summary of Results. GMC, 2020.

[21] Greig F. COVID-19, medical education and the impact on the future psychiatric workforce. BJPsych Bull 2021; 45: 179–83. 33261711 10.1192/bjb.2020.112 PMC7711332

[22] Blencowe NS, Van Hamel C, Bethune R, Aspinall R. ‘From scared to prepared’: targeted structured induction training during the transition from medical school to foundation doctor. Perspect Med Educ 2015; 4: 90–2. 25870119 10.1007/s40037-015-0168-x PMC4404456

[23] Dave MS, Mobarak S, Spiers HVM, Tarazi M, Jamdar S. Improving knowledge and confidence in foundation doctors during specialty changeover. Int J Qual Health Care 2020; 32: 490–4. 32671391 10.1093/intqhc/mzaa070

[24] Banerjee D, Rai M. Social isolation in Covid-19: the impact of loneliness. Int J Soc Psychiatry 2020; 66: 525–7. 32349580 10.1177/0020764020922269 PMC7405628

[25] Lane V, White J, Lane M. Situational on call survival (SOS) guide – qualitative assessment of a novel peer induction initiative. MedEdPublish 2016; 5(2): 10.

[26] Stone S, Stark D, Bullock C. PG119 Adapting on-call simulation for final year medical students to an online format during the Covid-19 pandemic. BMJ Simul Technol Enhanced Learn 2020; 6(Suppl 1): A97.

[27] Beal MD, Kinnear J, Anderson CR, Martin TD, Wamboldt R, Hooper L. The effectiveness of medical simulation in teaching medical students critical care medicine: a systematic review and meta-analysis. Simul Healthcare 2017; 12: 104–16. 10.1097/SIH.0000000000000189 28704288

[28] Semler MW, Keriwala RD, Clune JK, Rice TW, Pugh ME, Wheeler AP, et al. A randomized trial comparing didactics, demonstration, and simulation for teaching teamwork to medical residents. Ann Am Thorac Soc 2015; 12: 512–9. 25730661 10.1513/AnnalsATS.201501-030OC PMC5466182

[29] Maloney S, Haines T. Issues of cost-benefit and cost-effectiveness for simulation in health professions education. Adv Simul 2016; 1(1): 13. 10.1186/s41077-016-0020-3 PMC5806357 29449982

[30] Fletcher JD, Wind AP. Cost considerations in using simulations for medical training. Mil Med 2013; 178: 37–46. 10.7205/MILMED-D-13-00258 24084304

[31] Zigman D, Young M, Chalk C. Using simulation to train junior psychiatry residents to work with agitated patients: a pilot study. Acad Psychiatry 2013; 37: 38–41. 23338872 10.1176/appi.ap.11070129

[32] Naik VN, Brien SE. Review article: simulation: a means to address and improve patient safety. Can J Anaesth 2013; 60: 192–200. 23239487 10.1007/s12630-012-9860-z

